# Characterizing partial AZFc deletions of the Y chromosome with amplicon-specific sequence markers

**DOI:** 10.1186/1471-2164-8-342

**Published:** 2007-09-28

**Authors:** Paulo Navarro-Costa, Luísa Pereira, Cíntia Alves, Leonor Gusmão, Carmen Proença, Pedro Marques-Vidal, Tiago Rocha, Sónia C Correia, Sónia Jorge, António Neves, Ana P Soares, Joaquim Nunes, Carlos Calhaz-Jorge, António Amorim, Carlos E Plancha, João Gonçalves

**Affiliations:** 1 Centro de Genética Humana, Instituto Nacional de Saúde Dr. Ricardo Jorge, 1649-016 Lisboa, Portugal; 2 Unidade de Biologia da Reprodução, Instituto de Medicina Molecular, Faculdade de Medicina de Lisboa, 1649-028 Lisboa, Portugal; 3 IPATIMUP – Instituto de Patologia e Imunologia Molecular da Universidade do Porto, 4200-465 Porto, Portugal; 4 Faculdade de Medicina da Universidade do Porto, 4200-319 Porto, Portugal; 5 Unidade de Nutrição e Metabolismo, Instituto de Medicina Molecular, Faculdade de Medicina de Lisboa, 1649-028 Lisboa, Portugal; 6 Unidade de Medicina da Reprodução, Maternidade Dr. Alfredo da Costa, 1069-089 Lisboa, Portugal; 7 Unidade Pluridisciplinar de Reprodução Humana, Hospital de Santa Maria, 1649-028 Lisboa, Portugal; 8 Faculdade de Ciências da Universidade do Porto, 4169-007 Porto, Portugal

## Abstract

**Background:**

The AZFc region of the human Y chromosome is a highly recombinogenic locus containing multi-copy male fertility genes located in repeated DNA blocks (amplicons). These AZFc gene families exhibit slight sequence variations between copies which are considered to have functional relevance. Yet, partial AZFc deletions yield phenotypes ranging from normospermia to azoospermia, thwarting definite conclusions on their real impact on fertility.

**Results:**

The amplicon content of partial AZFc deletion products was characterized with novel amplicon-specific sequence markers. Data indicate that partial AZFc deletions are a male infertility risk [odds ratio: 5.6 (95% CI: 1.6–30.1)] and although high diversity of partial deletion products and sequence conversion profiles were recorded, the AZFc marker profiles detected in fertile men were also observed in infertile men. Additionally, the assessment of rearrangement recurrence by Y-lineage analysis indicated that while partial AZFc deletions occurred in highly diverse samples, haplotype diversity was minimal in fertile men sharing identical marker profiles.

**Conclusion:**

Although partial AZFc deletion products are highly heterogeneous in terms of amplicon content, this plasticity is not sufficient to account for the observed phenotypical variance. The lack of causative association between the deletion of specific gene copies and infertility suggests that AZFc gene content might be part of a multifactorial network, with Y-lineage evolution emerging as a possible phenotype modulator.

## Background

The human Y chromosome contains relatively few genes but exhibits remarkable functional coherence since many of them are directly or indirectly related to sex determination and fertility [1]. Approximately 10 megabases (Mb) of the Y consists of complex arrays of individual repeating units (designated as amplicons), each spanning up to 700 kilobases (kb) [2]. Amplicons are divided in different families, each one possessing very high sequence identity between member copies (99.8%) [2,3]. It has been established that all genes with testis-specific or predominant expression, except *SRY*, locate to these units and are consequently arranged in multi-copy gene families [2]. Yet, genome architectures based on repetitive units favour the occurrence of nonallelic homologous recombination (NAHR), leading to both chromosome duplications and corresponding deletions [4]. Accordingly, both Y duplications and deletions have been reported, the latter convincingly associated to male infertility [5].

In fact, these deletions were crucial for mapping three different regions required for spermatogenesis in the long arm of the Y (Yq). They were termed AZFa, b and c (for AZoospermia Factor), with complete deletions of AZFa or AZFb corresponding to a well-defined phenotype [6]. Although the complete AZFc deletion leads to mixed germ cell atrophy and hypospermatogenesis, AZFc gene function and regulation remain largely unknown as illustrated by the rare cases of natural transmission of such deletions from father to sons [7-11]. AZFc is composed almost in its entirety of amplicons, with the reference sequence being organized in five families, each with copy numbers ranging from two to four [3]. This arrangement, by packing together highly similar sequence units in a 3.5 Mb contiguous genomic stretch, favours the occurrence of NAHR between amplicon copies belonging to the same sequence family. Accordingly, several partial AZFc deletions resulting from recombination between internal AZFc amplicons have already been described [12-16]. These partial deletions are associated to extremely variable phenotypes, ranging from normo to azoospermia, making definite conclusions on their real impact for male fertility a source of controversy [17-20]. In addition, partial deletion rates also vary considerably between populations, with some being fixed with no apparent effect on fertility in several Y-lineages, further confounding association studies [13-15]. However, the source of the variable phenotypes attributed to partial deletions may lie in the specificities of AZFc's structure.

Large-scale variations of AZFc architecture as a result of inversions and duplications/deletions are estimated to occur at particularly elevated levels [21]. By adding frequent gene conversions [22], AZFc's variability rate may reach an unprecedented scale for a human non-satellite locus. This implies that the available AZFc reference sequence may only represent a fraction of the plethora of possible rearrangements. Therefore, partial AZFc deletions by resulting from variable amplicon recombinations and by occurring on extremely diverse AZFc structural backgrounds may result in a heterogeneous pool of deletion products with varying gene copy content. Since human duplicate genes have been shown to diverge rapidly in their spatial expression [23], it has been proposed that the different copies of AZFc genes vary in terms of functional properties [12,16]. Thus, characterizing exactly which copies remain in partially deleted chromosomes of fertile and infertile men may explain the variable phenotypes associated to these deletions and might shed new light on the functional roles of the various copies of AZFc genes.

In this study, we characterized with novel amplicon-specific markers the AZFc amplicon content and Y-lineage of men with partial deletions and of a sample of the fertile and infertile male population, in order to identify the extent of AZFc diversity and detect evidence of functional variance between gene copies.

## Results

### Partial AZFc deletions, as detected by an AZFc sequence tagged site (STS) panel, are a heritable male infertility risk

An initial screening for partial AZFc deletions in both fertile and infertile men was performed using a previously published amplicon-boundary STS panel. Partial AZFc deletions were significantly more frequent in infertile men when compared to fertile men: 16/300 (5.3%) *vs*. 3/300 (1.0%; *P *< 0.005). This accounted for an odds ratio of 5.6 (95% CI: 1.6–30.1) of possessing a partial deletion and being infertile. In the infertile group, partial deletions were recorded at similar rates both in azoospermic (5/90; 5.6%) and oligozoospermic men (11/210; 5.2%; *P *> 0.05). Out of the 4 fathers analysed, half of the partial deletions were *de novo*.

Eighteen of the 19 detected partial deletions corresponded to absence of amplification of sY1291 (previously reported as the gr/gr deletion) and one to sY1192 (gr/gr deletion after b2-b3 inversion, or b2/b3 deletion after a gr-rg inversion). In the 15 patients analysed by *Eco*RV DNA blotting with the 49f probe, results indicated that absence of amplification of the STSs was always associated with reduction in *DAZ *gene copy number, thereby confirming the deletions [see Additional file 1].

### AZFc sequence conversions are observed with similar frequencies in fertile and infertile men

The AZFc region of 50 idiopathic infertile and 50 fertile men, both without partial deletions as assessed by a previous STS screening, was characterized using novel amplicon-specific sequence family variants (SFVs) and STS. Since this group consisted of men with no partial deletions, the absence of any amplicon-specific variant was assumed to be the result of sequence conversion. The informativity of the novel SFVs was confirmed by sequencing the full extent of all markers in 30 men from various Y-haplogroups and comparing the data to the original reference sequence used as template. The SFVs were considered informative since all nucleotide variations matched those expected from the reference sequence. Conversions of the reference AZFc sequence were detected in 37 men (37%), 20 of which were fertile (54%), and the remaining 17 infertile (46%). Globally, 87 conversion events were recorded in 37 men, with thirteen different conversion profiles detected: seven corresponding to single conversions while the other six were associated to multiple conversions [see Additional file 1]. Moreover, the detected conversion patterns reflected the differences between Y-lineages, with conversions restricted to the loss of single variants in haplogroup R whereas in haplogroups E, J and I multiple conversions were largely predominant.

### Partial AZFc deletions correspond to diverse amplicon-specific marker profiles with varying gene content

The AZFc amplicon content of men with partial deletions as detected in the initial STS screening was ascertained by analysing amplicon-specific SFV/STS profiles. This analysis served to identify gene copy deletions and partial deletion sub-types. Although data from the preliminary STS screening indicated that all the 19 deletions were gr/gr deletions (even though one case could also correspond to a b2/b3 deletion profile), the analysis of the amplicon-specific markers revealed high sequence variability, as reflected in 14 different marker profiles with varying gene content [see Additional file 1].

**Figure 1:**
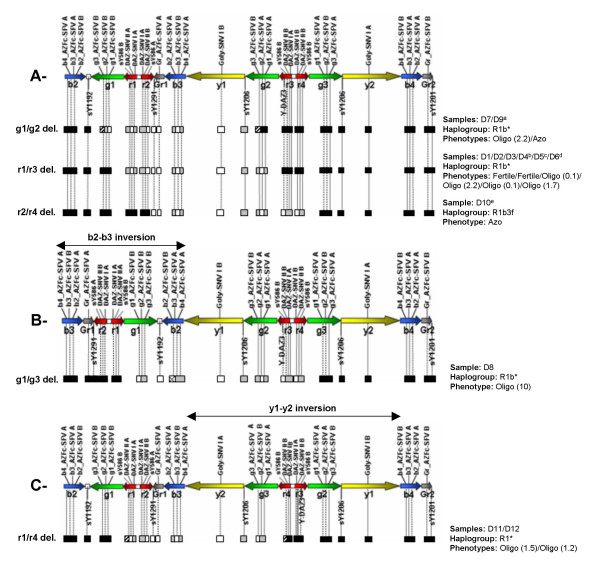
Partial AZFc deletion sub-types in samples belonging to haplogroup R (reference sequence). The blue (b), green (g), red (r) and yellow (y) families contain multiple copies of the *PRY*, *BPY2*, *DAZ *and *CDY1 *genes, respectively, with purportedly different functional capabilities between copies. Black box: present region; white box: absent region; grey box: multiple-copy marker that is considered absent in that specific position according to the deletion context; diagonal lines box: multiple-copy marker for which data is not sufficient to confirm either presence or absence; crossed box: marker present due to previous inversion event occurring between the b2 and b3 amplicons. Letters A-C denote the AZFc background where the deletions occurred. **A- **Reference AZFc sequence. **B- **b2-b3 inversion in the reference AZFc sequence, as previously described. **C- **y1-y2 inversion in the reference AZFc sequence. This AZFc background is proposed as the most parsimonious for the observed deletion products, yet no fluorescence *in situ *hybridization confirmation of this rearrangement is possible due to the symmetry of the inversion. Average sperm count of oligozoospermic men is indicated in parentheses (in million sperm/ml). ^a^- Sample with additional sequence conversions in the b2 and g2 markers. ^b^- The deletion breakpoint (mapped between exons 4 and 10 of *DAZ1*/*DAZ3*) is more distal than in the 3 previous samples. ^c^- Sample with sequence conversion leading to the loss of the b2-specific variant. ^d^- Sample with sequence conversion leading to the loss of the g3-specific variant. ^e^- Sample with sequence conversion leading to the loss of the b4-specific variant. [For complete marker profiles and full haplogroup nomenclature please see Additional file 1].

The assignment of deletion sub-types to each of these profiles was only possible in samples belonging to haplogroup R (the available reference sequence). In these patients, a total of four different amplicon recombinations could be assigned as the expected gr/gr deletion sub-types: g1/g2, r1/r3, r2/r4 and r1/r4, with the latter requiring a putative y1-y2 inversion before the deletion step (Figure 1). In the haplogroup R sample with the absent sY1192 STS, results were in accordance to the previously described b2-b3 inversion followed by a gr/gr deletion, with our data indicating a g1/g3 deletion sub-type (Figure 1). This type of analysis was not possible for deleted samples belonging to other haplogroups, since the marker profiles were incompatible with the amplicon architecture observed in the available reference sequence, suggesting different AZFc backgrounds [see Additional file 1].

### Fertile men with partial AZFc deletions have identical amplicon-specific marker profiles to infertile men

Deletions of the r1, r2, b3, y1, g2, r3 and r4-specific SFVs were recorded in fertile men with partial AZFc deletions. These corresponded to the loss of specific copies of the *DAZ*, *CDY1 *and *BPY2 *gene families. Yet, all AZFc amplicon-specific marker profiles recorded in fertile men were also identified in infertile patients [see Additional file 1]. Specifically, the two fertile men belonging to haplogroup R had marker profiles identical to those of two infertile patients with severe spermatogenic impairment (average of 2.2 and 0.1 million sperm/ml). This trend was equally observed in the three men from haplogroup E sharing identical marker profiles. Interestingly, one was fertile, the other oligozoospermic (average of 6.0 million sperm/ml) and the third was azoospermic. As for patients belonging to haplogroups J and I, no fertile men nor identical marker profiles were observed to allow phenotype comparisons.

### Although partial AZFc deletions occur in very diverse Y-lineages, haplotype diversity is reduced in fertile men with identical amplicon-specific marker profiles

The Y chromosome paragroup, haplogroup and haplotype of men with partial AZFc deletions were characterized in order to identify deletion recurrence and the evolutionary diversity between individuals belonging to the same Y-haplogroup and sharing identical AZFc compositions. All 19 partial AZFc deletions were detected in patients belonging to the predominant Y-haplogroups of the Portuguese population: R (12/19 cases), E (4/19), J (2/19) and I (1/19). Although the small sample size precluded the use of association tests, this sample could largely be considered as similar to a standard Y-haplogroup distribution of the studied population. Intra-haplogroup deletion recurrence was detected in paragroups R [R1b1*(xR1b1a, b, c1, c2, c5, c6)-P25, R1*(xR1a, b1)-M173 and R1b1c6-SRY_2627_] and J [J*(xJ1,2) -12f2.1 and J2-M172], but not in haplogroups E [all samples were E3b1*(xE3b1a, c1)-M35 and I [only one sample, I*(xI1b1b)-M170].

The microsatellite analysis indicated high haplotype diversity between individuals sharing identical AZFc marker profiles and Y-haplogroups. This diversity, measured by the mean number of pairwise differences (MNPDs) between samples, although not statistically significant, could surpass the baseline value calculated for the tested population, as observed in three individuals of sub-haplogroup E3b1*(xE3b1a, c1) with MNPDs of 4.0 ± 2.7 against a population baseline of 2.8 ± 1.5. Interestingly, when the two fertile men with identical marker profiles belonging to sub-haplogroup R1b1*(xR1b1a, b, c1, c2, c5, c6) were compared, they differed only in one single step mutation.

## Discussion

The effects of partial AZFc deletions on male fertility are at the source of several conflicting reports [16-20,24-27]. Partial AZFc deletions were shown not to be exclusively associated to abnormal sperm concentrations but significantly so to infertility [17,19,20,25,27,28], thus selecting controls based solely in normal sperm counts may be a considerable confounding factor. However, since fertility reflects a combination of male and female factors, both semen analysis and female partner evaluation are important aspects for the selection of the fertile population, the lack of which have to be considered as limitations to this study. Another key issue to be taken into account is that the outcome of association tests is heavily dependent on the Y-haplogroup distribution of the sampled population. Partial AZFc deletions are fixed in haplogroups D2b, N and Q with no apparent effect on fertility [13-15,28], as a probable consequence of haplogroup-specific compensatory factors. Consequently, tests performed in populations in which these lineages have significant prevalence will be underpowered to detect any putative partial deletion effect in other haplogroups. Therefore, using fertile men as controls and characterizing the Y-lineages of the studied population in order to rule out partially-deleted haplogroup enrichment give novel and complementing insight to the analyses published thus far. In this study, partial AZFc deletions were associated to male infertility with an odds ratio of 5.6 (95% CI: 1.6–30.1). The observation that 1% of fertile men carry this deletion is consistent with the detection of paternal transmission, suggesting that a subset of partial AZFc deletions is compatible with fertility. In fact, differences in length and/or deleted copy identity could explain the variable phenotypes associated to these deletions.

In this context, expanding the resolution of the original AZFc STS panel via the use of additional amplicon-specific markers provided the necessary power to differentiate AZFc deletion products. Although fluorescent *in situ *hybridization (FISH) protocols and quantitative PCRs can be used for visualizing amplicon order and copy number variation, only sequence-based qualitative analyses can accurately differentiate between copy family members and reveal the purported diversity of gene content between partial deletions. Actually, due both to their reproducibility and aptness for large cohort studies, the use of SFVs for the clinical molecular characterization of such deletions is an emerging trend [27,29]. Yet to be fully informative, amplicon-specific markers require Y-lineage characterization and partial deletion STS screening to avoid the previously demonstrated pitfalls of conversion-mediated variant loss [30]. Accordingly, our results indicate that the high levels of sequence conversion in non-deleted AZFc regions reflect the evolutionary differences between Y-lineages, with single conversions preferentially detected in haplogroup R and conversions affecting multiple variants solely recorded in haplogroups E, J and I. This indicates that sequencing AZFc in different haplogroups may yield crucial information on the evolution and functional specialization of amplicon gene copies and that clinical use of SFVs requires previous characterization of the sequence conversion profiles present in the sampled population to avoid erroneous interpretation of variant losses.

The recombinogenic potential of AZFc was clearly patent in the 14 different marker profiles recorded in 19 men with partial deletions. By considering the simultaneous action of deletion/duplications, inversions and sequence conversions, diverse AZFc deletion products could be expected in the male population. In fact, this study demonstrates for the first time the extent of AZFc sequence variability in a population sub-set of clinical importance. Although the present data indicates that partial AZFc deletions correspond to a heterogeneous pool of AZFc architectures with varying gene content, amplicon-specific marker profiles detected in fertile men were also recorded in oligo and azoospermic patients, compromising the establishment of robust genotype-phenotype correlations. Interestingly, our data indicate that the y1 and g2 copies of *CDY1 *and *BPY2*, respectively, are not essential for fertility since deletions of both were detected in fertile men. Deletions of *DAZ1 *and *DAZ2*, as well as of *DAZ3 *and *DAZ4 *were also detected in fertile men, suggesting some functional overlap between the *DAZ *gene copies.

The unexpected lack of genotype-phenotype correlation can be attributed to sequence differences in non-analysed AZFc domains, but can also raise the hypothesis that AZFc gene content might be part of a multifactorial network. This hypothesis is reinforced by the lack of causative association between the deletion of specific gene copies and infertility in our sample. Since it has been demonstrated that Y-lineages are structurally polymorphic [21], interplay between AZFc and Y chromosome background may play a crucial role in determining the phenotype associated to specific architectures. In the present study, even though partial deletions were detected in highly diverse haplotypes, the haplotype match in the two fertile males most probably indicates that such chromosomes are identical by descent (although they were not directly related), which could support the hypothesis of a haplotype protective effect. This hypothesis, if corroborated by future large-scale studies, would imply that partial deletions with no discernible effects on fertility may occur in specific Y backgrounds with compensatory mechanisms alleviating deletion effects. Seeing that partial AZFc deletions remove a considerable fraction of Yq and have been linked both to disturbances in Xq-Yq telomere pairing and segregation deficiencies [31-33], the variable length of the distal heterochromatin block between Y-lineages may be a candidate feature modulating the deletion's phenotypical expression.

## Conclusion

This study demonstrates that partial AZFc deletions correspond to a clear male infertility risk in the selected population. Furthermore, it was identified that AZFc amplicon content varies widely in the partial AZFc deletion pool, but specific genetic variants are still associated to variable phenotypes. As identical amplicon-specific marker profiles were detected in very diverse haplotypes, the possibility of the observed phenotypical variance being modulated by lineage-specific evolutionary mechanisms is proposed. The use of these amplicon-specific markers to fully characterize AZFc sequence plasticity in worldwide Y-lineages may lead to a better understanding of the functional basis responsible for AZFc-mediated male infertility.

## Methods

### Study population

To accurately assess the effects of partial AZFc deletions on male fertility, a group of 300 men of proven fertility attending our Centre for prenatal diagnosis or family studies of Mendelian traits [type and percentages of traits: carriers of mutations in *HBB *gene involved in pre-natal diagnosis of β thalassaemia (31%), non-affected fathers of patients with mutations in the *CYP21A2 *gene (19%), non-affected fathers of patients with or without mutations in the *CFTR *gene (18%), fathers of children with neuroblastoma (14%), non-affected fathers of patients with haemophilia A and B (6%), non-affected or carrier family members of other various conditions present in small numbers (12%)] were retrospectively selected as controls (only one father from each family was included, with no data on sperm counts). For the infertile group, another 300 men were selected from the Centre's male infertility DNA bank. Selection criteria included: idiopathic infertility, sperm counts below 10 × 10^6 ^sperm/ml irrespective of sperm morphology and motility (more than two spermograms per patient), normal karyotype and absence of AZF microdeletions (present STSs: DBY, DFFRY, RBMY1, sY1224, sY134, sY143, sY119, sY283, RRM3 and sY254). The 300 infertile patients corresponded to 210 men with oligozoospermia and 90 with non-obstructive azoospermia. The selected groups were considered representative of the male Portuguese population since the Centre is a central reference laboratory receiving samples on a nationwide scale.

All patients gave their written informed consent to all analyses, and the study was performed in compliance with the Helsinki Declaration and according to the guidelines of the Scientific and Ethics Committee of the Centre of Human Genetics of the National Institute of Health, Lisbon.

### STS screening of partial AZFc deletions

**Figure 2:**
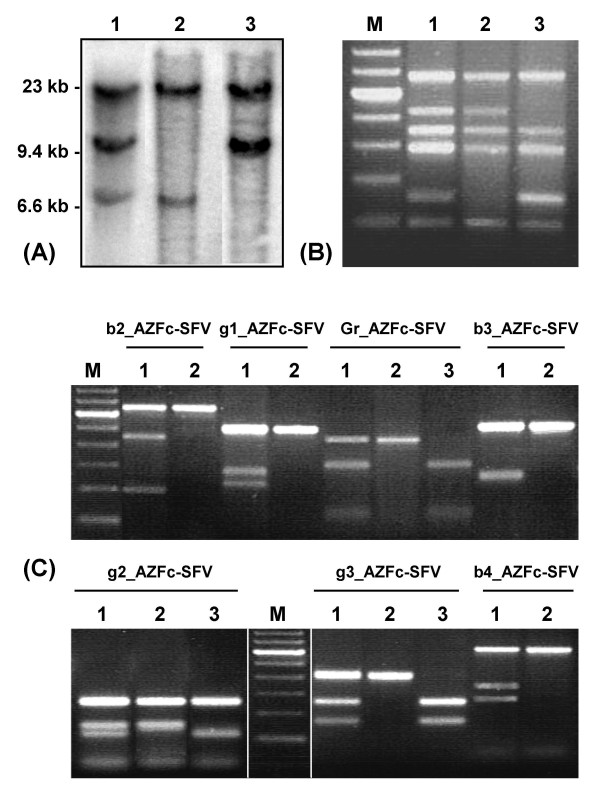
DNA blots and PCR assays for the detection of AZFc rearrangements. **(A)**- *Eco*RV DNA blot analysis with the 49f probe (DYS1). The deletion of *DAZ1 *(specific for amplicon r1) and *DAZ4 *(amplicon r4) are indicated by absence of the 10.8 and 7.3 kb fragments, respectively. **1**- Control sample (no deletions). **2**- *DAZ1 *deletion. **3**- *DAZ4 *deletion. **(B)**- AZFc STS panel for the detection of partial deletions: sY1201 (677 bp), sY1291 (527 bp), sY1197 (453 bp), sY1206 (394 bp), sY1192 (255 bp), and sY142 (196 bp). **1**- Control sample (no deletions). **2**- sY1192 negative (gr/gr deletion after b2-b3 inversion, or b2/b3 deletion after a gr-rg inversion). **3**- sY1291 negative (gr/gr deletion). **M**- 100 bp DNA marker. Higher intensity band corresponds to 600 bp. **(C)**- Novel sequence-family variants (SFVs) digestion profiles. For each SFV: **1**- Allele A+B. **2**- Allele A. **3**- Allele B. For fragment size and allele amplicon specificity please consult Table 1. **M**- 100 bp DNA marker. Higher intensity band corresponds to 600 bp.

Partial AZFc deletion screening was performed in genomic DNA samples using a previously published STS panel [13]: sY142, sY1197, sY1192, sY1291, sY1206 and sY1201 (Figure 2). Whenever partial deletions were detected in the fertile group, paternal allele transmission to the progeny was further reconfirmed by typing 9 autosomal short tandem repeat loci according to the manufacturers' instructions (AmpFLSTR Profiler Plus; Applied Biosystems, Foster City, USA). To test for the transmission of partial AZFc deletions, authorization to obtain a paternal blood sample in infertile men with such deletions was requested. Four samples were thus obtained and screened as above.

**Table 1:** Novel AZFc amplicon-specific sequence family variants (SFVs)

SFV	GenBank accession number	AZFc reference assembly BAC mapping^a, b^	SFV position (bp in each BAC)	Restriction Enzyme	Restriction Fragments (bp)	Allelic variants^c^
b2_AZFc-SFV	BV686548	AC008175 (b2)	62.881–63.530	*Mnl*I	A- 653	+3nt^d ^(b1, b3, b4)
		AC016752 (b3)	80.268–80.920		B- 452+197	no insertion (b2)
		AC007965 (b4)	99.077–99.729			
b3_AZFc-SFV	BV686549	Same as above	52.143–52.652	*Bln*I	A- 510	T (b1, b2, b4)
			91.161–91.670		B- 261+249	C (b3)
			88.327–88.836			
b4_AZFc-SFV	BV686550	Same as above	43.853–44.482	*Hph*I	A- 630	G (b1, b2, b3)
			99.325–99.954		B- 347+282	A (b4)
			80.041–80.670			

g1_AZFc-SFV	BV686551	AC006366 (g1)	59.421–59.920	*Bsm*AI	A- 500	C (g2, g3)
		AC010153 (g2)	54.938–55.437		B- 272+224	G (g1)
		AC016728 (g3)	50.071–50.570			
g2_AZFc-SFV	BV686552	Same as above	61.496–61.987	*Tsp*45I	A- 274+170+38	G (g2)
			52.871–53.362		B- 274+137+38+28	C (g1, g3)
			52.146–52.637			
g3_AZFc-SFV	BV686553	Same as above	62.357–62.774	*Bsm*AI	A- 400	no insertion (g3)
			52.084–52.501			
			53.007–53.406		B- 251+163	+18nt^e ^(g1, g2)

Gr_AZFc-SFV	BV686554	AC006983 (Gr1)	158.663–159.102	*Sna*BI	A- 440	A (Gr1)
		AC024067 (Gr2)	13.026–13.465		B- 311+129	G (Gr2)

a- GenBank accession numbers corresponding to the BAC clones containing the SFV copies (amplicon in parentheses).

b- The blue amplicon family SFVs (b2_AZFc-SFV, b3_AZFc-SFV and b4_AZFc-SFV) also map to AC007359 (b1).

c- Amplicon(s) specific for each allele are represented in parentheses.

d- 462_463insAAG e- 153_154insGAGAGTCTCATCACCTGG

### Confirmation of partial AZFc deletions by DNA blotting

DNA blotting was performed in *Eco*RV-digested genomic DNA samples from 15 patients with partial AZFc deletions according to a previously published protocol [12]. Radioactive probing was performed with the 2.8 kb *Eco*RI fragment of plasmid p49f, previously mapped to the region extending from the third RRM domain to exon 7B of the *DAZ1 *gene copy [12]. Visualization of hybridization signal associated to the 10.8 kb and 7.3 kb genomic bands (specific for *DAZ1*, located in amplicon r1; and *DAZ4*, in amplicon r4, respectively) was performed by autoradiography (Figure 2).

### SFV/STS profiling of the AZFc region

An amplicon-specific marker profiling was performed in all men with partial deletions (n = 19), their ascendants (n = 4), and a sample of men without partial AZFc deletions, as detected by STSs screening (n = 100, consisting of 50 idiopathic infertile and 50 fertile men as previously defined). The selected amplicon-specific markers included all the novel SFVs [for information on SFV design see Additional file 2]: b2_AZFc-SFV, g1_AZFc-SFV, Gr_AZFc-SFV, b3_AZFc-SFV, g2_AZFc-SFV, g3_AZFc-SFV and b4_AZFc-SFV [GenBank:BV686548, BV686551, BV686554, BV686549, BV686552, BV686553 and BV686550, respectively] (Table 1); as well as previously published markers: DAZ-SNV II, sY586, DAZ-SNV I and Goly-SNV I [GenBank:G73166, G63907, G73167 and BV012733], specific for r1, r2, r4 and the yellow amplicons respectively, and Y-DAZ3 [GenBank:G73170], specific for r3. PCR product digestions were performed with the appropriate restriction enzymes according to the suppliers' instructions. Digestions were analysed in 2% agarose gels (1 LE: 1 NuSieve), stained with ethidium bromide (Figure 2).

### Y-lineage characterization

An adaptation of the previously described four multiplexes SNaPshot strategy was followed for haplogroup assignment in samples with non-reference marker profiles [34]. Multiplex 1, consisting of 9 markers (M22, P25, SRY10831, 92R7, M173, M70, Tat, M213 and M9), was typed in all samples, allowing a broad haplogroup classification, depending on which the samples were typed either for Multiplex 2 (6 markers: 12f2.1, M170, M62, M172, M26, M201), Multiplex 3 (4 markers: M34, M78, M35, M96), or Multiplex 4 (7 markers: SRY2627, M17, M18, M37, M73, M65, M160). For haplotyping purposes, the 17 Y-chromosomal STRs included in the AmpF/STR^® ^Yfiler™ PCR Amplification kit (Applied Biosystems; Foster City, USA) were screened according to the manufacturers' instructions. Nomenclature followed the criteria of Y Chromosome Consortium 2002 [35] (updated for haplogroups E [36], R [37] and I [38]) and the International Society of Forensic Genetics guidelines [39].

### Statistical analysis

Partial deletion frequencies in the patient and control groups were compared using the chi square and odds ratio Tests on SPSS v14.0 (SPSS Inc., Chicago, USA) with *P *values < 0.05 statistically significant. The pairwise genetic distances between groups of haplotypes was assessed by the *R*_ST _Test using the Arlequin software v2.0 [40], and tested for statistical significance by means of randomisation (1,000 replicates per comparison). Results are presented as MNPDs between samples. For comparison purposes, the baseline MNPDs assigned to specific haplogroups of a reference Portuguese population sample were determined [41].

## Competing interests

The author(s) declares that there are no competing interests.

## Authors' contributions

PNC performed the STS screening, amplicon-specific marker profiling and DNA blotting, participated in the sequence alignment and drafted the manuscript. LP, CA and LG performed the Y-lineage assays and drafted the respective sections of the manuscript. CP performed additional amplicon-specific marker profiling. PMV participated in study design and performed the statistical analyses. TR, SCC, SJ, APS and JN performed the clinical characterization of the infertile men and were involved in patient selection. AN and CCJ coordinated patient selection and provided critical improvements to the manuscript. AA, CEP and JG conceived the study, participated in its design and coordination and helped to draft the manuscript. All authors read and approved the final manuscript.

## Supplementary Material

Additional file 1 AZFc marker profile table. The data provided correspond to the ampliconic-specific marker profiles of men with AZFc gene conversions and with partial AZFc deletions. Click here for file

Additional file 2 Amplicon-specific marker design methodology. Description of the chosen methodology for the design of novel AZFc amplicon-specific sequence family variants. Click here for file
