# Supplementary material for: Characterizing partial AZFc deletions of the Y chromosome with amplicon-specific sequence markers
Source: BMC Genomics. 2007 Sep 28;8:342. doi: 10.1186/1471-2164-8-342 (PMC2151955; doi:10.1186/1471-2164-8-342)
Supplement: Additional file 1 — AZFc marker profile table. The data provided correspond to the ampliconic-specific marker profiles of men with AZFc gene conversions and with partial AZFc deletions. [file 1471-2164-8-342-S1.doc]

| **Additional file 1** | | | | | | | | | | | | | | | | | | | | |
| --- | --- | --- | --- | --- | --- | --- | --- | --- | --- | --- | --- | --- | --- | --- | --- | --- | --- | --- | --- | --- |
| **Ampliconic-specific marker profiles in 37 men with AZFc gene conversions (C1-37) and 19 men with partial AZFc deletions (D1-19)** | | | | | | | | | | | | | | | | | | | | |
|  |  |  | 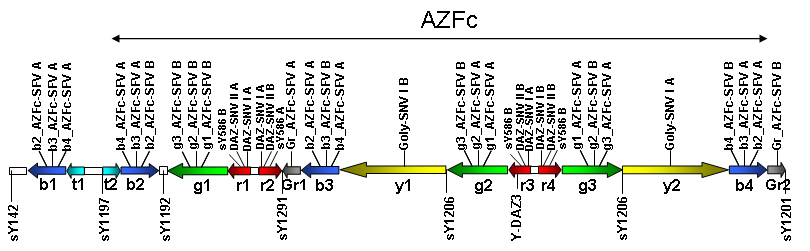 | | | | | | | | | | | | | | | | | |
|  |  |  | SFVs/STSs panel | | | | | | | | | | | | | | | | | |
| Sample code | Haplogroup^a^ | Phenotype^b^ | sY142^c^ | sY1197^c^ | b2_AZFc-SFV | sY1192 | g1_AZFc-SFV | DAZ_SNV II^d^  (*DAZ* DNA blot  result) | sY586 ^e^ | sY1291 | Gr_AZFc-SFV | b3_AZFc-SFV | Goly-SNV I^f^ | sY1206 | g2_AZFc-SFV | Y-DAZ3^g^ | DAZ_SNV I^h^  (*DAZ* DNA blot  result) | g3_AZFc-SFV | b4_AZFc-SFV | sY1201 |
| Ref^†^ | R1b3* | n/a | + | + | A+B | + | A+B | A+B | A+B | + | A+B | A+B | A+B | + | A+B | + | A+B | A+B | A+B | + |
| C1 | R1b1* | Fertile | + | + | A+B | + | A+B | A+B | A+B | + | A+B | A+B | A+B | + | **B** | + | A+B | A+B | A+B | + |
| C2 | R1b1* | Fertile | + | + | A+B | + | A+B | A+B | A+B | + | A+B | A+B | A+B | + | **B** | + | A+B | A+B | A+B | + |
| C3 | R1b1* | Infertile | + | + | A+B | + | A+B | A+B | A+B | + | A+B | A+B | A+B | + | **B** | + | A+B | A+B | A+B | + |
|  |  |  |  |  |  |  |  |  |  |  |  |  |  |  |  |  |  |  |  |  |
| C4 | R1b1* | Fertile | + | + | A+B | + | A+B | A+B | A+B | + | A+B | A+B | A+B | + | A+B | + | **A** | A+B | A+B | + |
| C5 | R1b1* | Fertile | + | + | A+B | + | A+B | A+B | A+B | + | A+B | A+B | A+B | + | A+B | + | **A** | A+B | A+B | + |
| C6 | R1b1* | Infertile | + | + | A+B | + | A+B | A+B | A+B | + | A+B | A+B | A+B | + | A+B | + | **A** | A+B | A+B | + |
| C7 | R1b1* | Infertile | + | + | A+B | + | A+B | A+B | A+B | + | A+B | A+B | A+B | + | A+B | + | **A** | A+B | A+B | + |
| C8 | R1b1* | Infertile | + | + | A+B | + | A+B | A+B | A+B | + | A+B | A+B | A+B | + | A+B | + | **A** | A+B | A+B | + |
| C9 | R1b1* | Infertile | + | + | A+B | + | A+B | A+B | A+B | + | A+B | A+B | A+B | + | A+B | + | **A** | A+B | A+B | + |
|  |  |  |  |  |  |  |  |  |  |  |  |  |  |  |  |  |  |  |  |  |
| C10 | R1b1* | Fertile | + | + | A+B | + | A+B | A+B | A+B | + | A+B | A+B | A+B | + | A+B | **-** | A+B | A+B | A+B | + |
| C11 | R1b1* | Infertile | + | + | A+B | + | A+B | A+B | A+B | + | A+B | A+B | A+B | + | A+B | **-** | A+B | A+B | A+B | + |
| C12 | R1b1* | Infertile | + | + | A+B | + | A+B | A+B | A+B | + | A+B | A+B | A+B | + | A+B | **-** | A+B | A+B | A+B | + |
|  |  |  |  |  |  |  |  |  |  |  |  |  |  |  |  |  |  |  |  |  |
| C13 | R1b1* | Infertile | + | + | A+B | + | A+B | A+B | A+B | + | A+B | A+B | **A** | + | A+B | + | A+B | A+B | A+B | + |
|  |  |  |  |  |  |  |  |  |  |  |  |  |  |  |  |  |  |  |  |  |
| C14 | R1b1* | Fertile | + | + | A+B | + | A+B | A+B | A+B | + | A+B | A+B | A+B | + | A+B | + | A+B | **B** | A+B | + |
| C15 | R1b1* | Fertile | + | + | A+B | + | A+B | A+B | A+B | + | A+B | A+B | A+B | + | A+B | + | A+B | **B** | A+B | + |
|  |  |  |  |  |  |  |  |  |  |  |  |  |  |  |  |  |  |  |  |  |
| C16 | E3b1a | Fertile | + | + | A+B | + | A+B | A+B | A+B | + | **A** | **A** | A+B | + | **B** | **-** | A+B | **B** | A+B | + |
| C17 | E3b1* | Fertile | + | + | A+B | + | A+B | A+B | A+B | + | **A** | **A** | A+B | + | **B** | **-** | A+B | **B** | A+B | + |
| C18 | E3b1a | Infertile | + | + | A+B | + | A+B | A+B | A+B | + | **A** | **A** | A+B | + | **B** | **-** | A+B | **B** | A+B | + |
| C19 | E3b1a | Infertile | + | + | A+B | + | A+B | A+B | A+B | + | **A** | **A** | A+B | + | **B** | **-** | A+B | **B** | A+B | + |
| C20 | E3b1* | Infertile | + | + | A+B | + | A+B | A+B | A+B | + | **A** | **A** | A+B | + | **B** | **-** | A+B | **B** | A+B | + |
| C21 | E3b1a | Infertile | + | + | A+B | + | A+B | A+B | A+B | + | **A** | **A** | A+B | + | **B** | **-** | A+B | **B** | A+B | + |
| C22 | E3b1* | Infertile | + | + | A+B | + | A+B | A+B | A+B | + | **A** | **A** | A+B | + | **B** | **-** | A+B | **B** | A+B | + |
|  |  |  |  |  |  |  |  |  |  |  |  |  |  |  |  |  |  |  |  |  |
| C23 | E3b1* | Fertile | + | + | A+B | + | A+B | A+B | A+B | + | A+B | **A** | A+B | + | **B** | **-** | **A** | **B** | A+B | + |
| C24 | E3b1a | Infertile | + | + | A+B | + | A+B | A+B | A+B | + | A+B | **A** | A+B | + | **B** | **-** | **A** | **B** | A+B | + |
|  |  |  |  |  |  |  |  |  |  |  |  |  |  |  |  |  |  |  |  |  |
| C25 | I* | Fertile | + | + | A+B | + | **A** | A+B | **B** | + | A+B | **A** | A+B | + | A+B | **-** | A+B | A+B | A+B | + |
|  |  |  |  |  |  |  |  |  |  |  |  |  |  |  |  |  |  |  |  |  |
| C26 | J2 | Fertile | + | + | **A** | + | **A** | A+B | **B** | + | **B** | **A** | A+B | + | A+B | + | A+B | A+B | A+B | + |
|  |  |  |  |  |  |  |  |  |  |  |  |  |  |  |  |  |  |  |  |  |
| C27 | J2 | Fertile | + | + | A+B | + | **A** | A+B | A+B | + | A+B | **A** | A+B | + | A+B | + | A+B | A+B | A+B | + |
| C28 | J2 | Fertile | + | + | A+B | + | **A** | A+B | A+B | + | A+B | **A** | A+B | + | A+B | + | A+B | A+B | A+B | + |
| C29 | J2 | Fertile | + | + | A+B | + | **A** | A+B | A+B | + | A+B | **A** | A+B | + | A+B | + | A+B | A+B | A+B | + |

*(Additional file 1 continues in the next page)*

|  |  |  |  |  |  |  |  |  |  |  |  |  |  |  |  |  |  |  |  |  |
| --- | --- | --- | --- | --- | --- | --- | --- | --- | --- | --- | --- | --- | --- | --- | --- | --- | --- | --- | --- | --- |
| C30 | J* | Infertile | + | + | A+B | + | **A** | A+B | A+B | + | A+B | **A** | **A** | + | A+B | + | A+B | A+B | A+B | + |
| C31 | J* | Infertile | + | + | A+B | + | **A** | A+B | A+B | + | A+B | **A** | **A** | + | A+B | + | A+B | A+B | A+B | + |
|  |  |  |  |  |  |  |  |  |  |  |  |  |  |  |  |  |  |  |  |  |
| C32 | J* | Infertile | + | + | A+B | + | **A** | A+B | A+B | + | A+B | A+B | A+B | + | A+B | + | A+B | A+B | A+B | + |
|  |  |  |  |  |  |  |  |  |  |  |  |  |  |  |  |  |  |  |  |  |
| C33 | R1b1* | Fertile | + | + | A+B | + | A+B | A+B | A+B | + | A+B | **A** | A+B | + | A+B | + | A+B | A+B | A+B | + |
| C34 | R1b1* | Fertile | + | + | A+B | + | A+B | A+B | A+B | + | A+B | **A** | A+B | + | A+B | + | A+B | A+B | A+B | + |
| C35 | G | Fertile | + | + | A+B | + | A+B | A+B | A+B | + | A+B | **A** | A+B | + | A+B | + | A+B | A+B | A+B | + |
| C36 | E3b1a | Fertile | + | + | A+B | + | A+B | A+B | A+B | + | A+B | **A** | A+B | + | A+B | + | A+B | A+B | A+B | + |
| C37 | J2 | Fertile | + | + | A+B | + | A+B | A+B | A+B | + | A+B | **A** | A+B | + | A+B | + | A+B | A+B | A+B | + |
|  |  |  |  |  |  |  |  |  |  |  |  |  |  |  |  |  |  |  |  |  |
| D1 | R1b1* | Fertile | + | + | A+B | + | A+B | **B (**○**)** | **B** | **-** | **B** | **A** | **A** | + | **B** | + | A+B (●) | A+B | A+B | + |
| D2 | R1b1* | Fertile | + | + | A+B | + | A+B | **B** | **B** | **-** | **B** | **A** | **A** | + | **B** | + | A+B | A+B | A+B | + |
| D3 | R1b1* | Oligo  (0.1 / 0.26) | + | + | A+B | + | A+B | **B (**○**)** | **B** | **-** | **B** | **A** | **A** | + | **B** | + | A+B (●) | A+B | A+B | + |
|  |  |  |  |  |  |  |  |  |  |  |  |  |  |  |  |  |  |  |  |  |
| D4 | R1b1* | Oligo  (2.2 / 6) | + | + | A+B | + | A+B | **B (**○**)** | **B** | **-** | **B** | **A** | **A** | + | **B** | **-** | A+B (●) | A+B | A+B | + |
|  |  |  |  |  |  |  |  |  |  |  |  |  |  |  |  |  |  |  |  |  |
| D5 | R1b1* | Oligo  (0.1 / 0.6) | + | + | **A** | + | A+B | **B (**○**)** | **B** | **-** | **B** | **A** | **A** | + | **B** | + | A+B (●) | A+B | A+B | + |
|  |  |  |  |  |  |  |  |  |  |  |  |  |  |  |  |  |  |  |  |  |
| D6 | R1b1* | Oligo  (1.7 / 5.5) | + | + | A+B | + | A+B | **B (**○**)** | **B** | **-** | **B** | **A** | **A** | + | **B** | + | A+B (●) | **B** | A+B | + |
|  |  |  |  |  |  |  |  |  |  |  |  |  |  |  |  |  |  |  |  |  |
| D7 | R1b1* | Oligo  (2.2 / 4) | + | + | A+B | + | **A** | **B** | **B** | **-** | **B** | **A** | **A** | + | A+B | + | A+B | A+B | A+B | + |
|  |  |  |  |  |  |  |  |  |  |  |  |  |  |  |  |  |  |  |  |  |
| D8 | R1b1* | Oligo  (10 / 27) | + | + | A+B | **-** | **A** | A+B (●) | A+B | + | A+B | A+B | **A** | + | **B** | **-** | **A (**○**)** | **A** | A+B | + |
|  |  |  |  |  |  |  |  |  |  |  |  |  |  |  |  |  |  |  |  |  |
| D9 | R1b1* | Azo | + | + | **A** | + | **A** | **B (**○**)** | **B** | **-** | **B** | **A** | **A** | + | **A** | + | A+B (●) | A+B | A+B | + |
|  |  |  |  |  |  |  |  |  |  |  |  |  |  |  |  |  |  |  |  |  |
| D10 | R1b1c6 | Azo | + | + | A+B | + | A+B | A+B (●) | A+B | - | **B** | **A** | **A** | **+** | **B** | **-** | **A (**○**)** | A+B | **A** | + |
|  |  |  |  |  |  |  |  |  |  |  |  |  |  |  |  |  |  |  |  |  |
| D11 | R1* | Oligo  (1.5 / 4.4) | + | + | A+B | + | A+B | **B** | **B** | **-** | **B** | **A** | **B** | + | A+B | + | A+B | **B** | A+B | + |
| D12 | R1* | Oligo  (1.2 / 2.6) | + | + | A+B | + | A+B | **B (**○**)** | **B** | **-** | **B** | **A** | **B** | + | A+B | + | A+B (●) | **B** | A+B | + |
|  |  |  |  |  |  |  |  |  |  |  |  |  |  |  |  |  |  |  |  |  |
| D13 | J* | Oligo  (0.1 / 0.25) | + | + | A+B | + | **A** | **B (**○**)** | **B** | **-** | **A** | **A** | **A** | + | A+B | + | A+B (●) | A+B | **A** | + |
|  |  |  |  |  |  |  |  |  |  |  |  |  |  |  |  |  |  |  |  |  |
| D14 | J2 | Azo | + | + | A+B | + | **A** | **A (**●**)** | **B** | **-** | **A** | **A** | **A** | + | **A** | **-** | **A (**○**)** | **B** | **A** | + |
|  |  |  |  |  |  |  |  |  |  |  |  |  |  |  |  |  |  |  |  |  |
| D15 | I* | Azo | + | + | A+B | + | **A** | **A (**●**)** | **B** | **-** | **B** | **A** | **B** | + | **B** | **-** | **A (**○**)** | **A** | **A** | + |
|  |  |  |  |  |  |  |  |  |  |  |  |  |  |  |  |  |  |  |  |  |
| D16 | E3b1* | Fertile | + | + | A+B | + | A+B | A+B | A+B | **-** | **A** | **A** | **A** | + | **B** | **-** | **A** | **B** | A+B | + |
| D17 | E3b1* | Oligo  (6 / 12.3) | + | + | A+B | + | A+B | A+B (●) | A+B | **-** | **A** | **A** | **A** | + | **B** | **-** | **A (**○**)** | **B** | A+B | + |
| D18 | E3b1* | Azo | + | + | A+B | + | A+B | A+B (●) | A+B | **-** | **A** | **A** | **A** | + | **B** | **-** | **A (**○**)** | **B** | A+B | + |
|  |  |  |  |  |  |  |  |  |  |  |  |  |  |  |  |  |  |  |  |  |
| D19 | E3b1* | Oligo  (3 / 6) | + | + | A+B | + | A+B | A+B (●) | A+B | **-** | **A** | **A** | **B** | + | **B** | **-** | **A (**○**)** | **B** | **A** | + |
| SFV results are coded by allelic designation (A, B or A+B; for allele specificity please consult Table 1), STSs by presence or absence of amplification (+/-) and *Eco*RV-49f DNA blots by presence or absence of the hybridizing bands (●/○).  All infertile men included in the conversion screening had idiopathic infertility and no complete or partial AZF deletions. Sperm counts were <10 million sperm/ml. Patients D3 and D4 have *de novo* deletions, while in patients D12 and D14 deletions were inherited.  a- Abbreviated haplogroup, where applicable. Full nomenclature according to references 35-38 follows. R1b1*: R1b1*(xR1b1a,b,c1,c2,c5,c6)-P25; R1*: R1*(xR1a,b1)-M173; E3b1*: E3b1*(xE3b1a,c1)-M35; J*: J*(xJ1,2)-M12f2.1; I*: I*(xI1b2)-M170.  b- Average sperm concentration (in million sperm per ml) / total sperm counts (in million sperm) of oligozoospermic men are indicated in parentheses.  c- STSs mapping to the AZFb region  d-h - Previously published markers specific for the following amplicons: d- r1, e- r2, f- y1/y2, g- r3, h- r4.  †- Reference sequence. This pattern was detected in 63 samples with no partial AZFc deletions. | | | | | | | | | | | | | | | | | | | | |
