# Supplementary material for: Characterizing partial AZFc deletions of the Y chromosome with amplicon-specific sequence markers
Source: BMC Genomics. 2007 Sep 28;8:342. doi: 10.1186/1471-2164-8-342 (PMC2151955; doi:10.1186/1471-2164-8-342)
Supplement: Additional file 2 — Amplicon-specific marker design methodology. Description of the chosen methodology for the design of novel AZFc amplicon-specific sequence family variants. [file 1471-2164-8-342-S2.doc]

**Additional file 2**

**Reference sequence analysis and amplicon-specific marker design**

Novel amplicon-specific markers for the b2, b3, b4, g1, g2, g3 and Gr1/Gr2 amplicons were designed by sequence analysis of the reference AZFc sequence, in order to detect subtle sequence variations (around 0.2%) between different amplicon copies belonging to the same family. This strategy allowed the design of 7 new amplicon-specific SFVs: b2_AZFc-SFV [GenBank:BV686548], g1_AZFc-SFV [GenBank:BV686551], Gr_AZFc-SFV [GenBank:BV686554], b3_AZFc-SFV [GenBank:BV686549], g2_AZFc-SFV [GenBank:BV686552], g3_AZFc-SFV [GenBank:BV686553] and b4_AZFc-SFV [GenBank:BV686550]; (Table 1). All *in silico* sequence homology analyses were performed using the CLUSTAL W multiple sequence alignment algorithm (European Bioinformatics Institute), with the Blosum30 matrix, full alignment and default gap settings [1]. To identify specific SFVs for the different members of the blue and green amplicon families, the reference sequences of the *PRY* gene (Entrez GeneID:  9081, sequence corresponding to the b4 amplicon copy) and TTTY4 transcript (Entrez GeneID:  114761, sequence corresponding to the g1 amplicon copy) were retrieved from the National Centre for Biotechnology Information and GenBank databases and used as probes in multiple sequence alignment rounds using as targets selected BAC subsets (containing the amplicon family members of the queried probe) derived from the reference assembly of the AZFc genomic contig [GenBank:NT_011903.11]. The results of the multiple alignments were manually compared after excluding variable-copy-number tandem repeats, allowing the selection of amplicon-specific nucleotide variations. Association of these variations with endonuclease restriction sites was tested using the Nebcutter v2.0 software in order to select markers with differential endonuclease restriction patterns [2]. A similar strategy was adopted for the analysis of the two grey amplicons (Gr1 and Gr2), but in this case the entire length of both amplicon copies was aligned since they contain no known genes or transcription units. Specifically, amplicon boundaries were mapped using the following STSs: Gr1 proximal: sY1291, distal: sY1125; Gr2 proximal: sY1125, distal: sY1201; and SFV design was performed as above.

To confirm the robustness of the novel SFVs, sequencing of all markers was performed in 30 samples representing the predominant Y-lineages of the sampled population (R, E, J, I) as well as some rarer haplogroups (G, K; typing and nomenclature described in the full manuscript). This corresponded to the analysis of a total of 109290 positions (3643 non-contiguous bases per individual). Sequencing was performed as previously published [3], and multiple sequences were aligned using the ChromasPro software (Technelysium Pty Ltd, Australia) with a CLUSTAL W plug-in in order to detect base substitutions. All putative mismatches were manually inspected by chromatogram analysis.

**References**

1 Thompson JD, Higgins DG, Gibson TJ: **CLUSTAL W: improving the sensitivity of progressive multiple sequence alignment through sequence weighting, position-specific gap penalties and weight matrix choice.** *Nucleic Acids Res* 1994, **22**:4673-4680.

2 Vincze T, Posfai J, Roberts RJ: **NEBcutter: a program to cleave DNA with restriction enzymes.** *Nucleic Acids Res* 2003, **31**:3688-3691.

3 Friaes A, Rego AT, Aragues JM, Moura LF, Mirante A, Mascarenhas MR, Kay TT, Lopes LA, Rodrigues JC, Guerra S, Dias T, Teles AG, Goncalves J: **CYP21A2 mutations in Portuguese patients with congenital adrenal hyperplasia: identification of two novel mutations and characterization of four different partial gene conversions.** *Mol Genet Metab* 2006, **88**:58-65.
